# Comparative Analysis of Organoleptic Preference and External Attractiveness of ‘Encore’ and ‘Nadorcott’ Mandarin Cultivars

**DOI:** 10.1155/ijfo/7941699

**Published:** 2026-05-24

**Authors:** Beatriz Duarte, Pedro Matias, Ana Rita Trindade, Amílcar Duarte

**Affiliations:** ^1^ MED–Mediterranean Institute for Agriculture, Environment and Development & Change–Institute for Global Change and Sustainability, Faculty of Science and Technology, Universidade do Algarve, Campus de Gambelas, Faro, 8005-139, Portugal, ualg.pt; ^2^ Faculty of Science and Technology, Universidade do Algarve, Campus de Gambelas, Faro, 8005-139, Portugal, ualg.pt; ^3^ CIMA/ARNET–Marine and Environmental Research Centre/Aquatic Research Network, Universidade do Algarve, Campus de Gambelas, Faro, 8005-139, Portugal, ualg.pt; ^4^ Higher Institute of Engineering, Universidade do Algarve, Campus da Penha, Faro, 8005-139, Portugal, ualg.pt

**Keywords:** citrus, consumer preference, external appearance, fruit quality, purchase intention, sensory analysis

## Abstract

The ‘Encore’ mandarin tree, cultivated in Portugal for long time, valued for its flavour and later ripening period, faces challenges due to a rind‐stain disorder affecting its external appearance. Despite its favourable attributes, the emergence of new cultivars such as ‘Nadorcott’, free from external appearance problems and with overlapping ripening periods, resulted in the marginalization of ‘Encore’ in the market. Major retailers, by prioritizing consumer preference for appearance, have contributed to the decline in ‘Encore’ cultivation. Despite this, the ‘Encore’ mandarin is still preferred by long‐standing consumers who remember its greater availability in previous decades and value its characteristic taste. Currently, its distribution is mainly restricted to local markets and small‐scale fruit retailers. To gauge consumer preference, we conducted a tasting and questionnaire survey with 131 randomly chosen participants, comparing ‘Encore’ and ‘Nadorcott’. The findings revealed a clear consumer preference for the external appearance of ‘Nadorcott’, leading to a preference for purchasing it based on this criterion alone. Conversely, when evaluating internal fruit quality, consumers distinctly favoured ‘Encore’ for its aroma, sweetness, acidity and overall taste. They expressed a preference for purchasing it based solely on internal quality. However, when participants learnt that less visually appealing fruits were equivalent to the more desirable ones internally, their purchase intentions became indifferent towards both cultivars, with no significant difference observed. This highlights the complex interplay between perceptions of external appearance and internal organoleptic quality among consumers, challenging the assumption that appearance alone dictates purchasing decisions.

## 1. Introduction

The Algarve, the southernmost region of Portugal, is the country’s main citrus‐producing area, primarily supplying the fresh fruit market and accounting for 87% of national orange production and 88% of national mandarin production [1]. It is the major agricultural activity in the region, which not only shapes the local landscape but also the diet and cultural habits of its communities [2, 3].

Among citrus fruits produced for fresh market, consumers are increasingly interested in mandarins due to their easy‐to‐peel nature [4] and flavour, which combines sweet, sour, fruity and fresh characteristics [5]. Seedlessness is also a crucial trait in mandarins as it significantly influences the ease and convenience of consumption [6].

The ‘Encore’ mandarin [*Citrus* × *nobilis*) × (*Citrus* × *deliciosa*)] holds significance in the Algarve citrus industry due to its organoleptic qualities, especially its highly appreciated flavour, and its niche position as one of few late‐season mandarin cultivars available [7–9]. Among many citrus cultivars, ‘Encore’ is one of the richest in total soluble solids (TSS), β‐carotene, total phenols and total flavonoids [10], although it exhibits one of the lowest vitamin C contents [11]. This cultivar is also prone to a preharvest physiological disorder known as rind‐stain (Figure 1(a)), which negatively impacts its visual appearance [8]. This disorder occurs mostly in rind areas exposed to solar radiation [12, 13]. The symptoms correspond to parenchymal flattening and progressive collapse of cell layers alongside the epidermis. The affected tissue collapses, dehydrates and develops small dark green spots, which gradually turn brown [8, 14]. Externally, this disorder resembles other rind blemishes induced by climatic conditions, mechanical injury, chemical treatments or postharvest chilling [8, 13]. Additionally, because ‘Encore’ mandarin is self‐compatible, it produces numerous seeds per fruit, an undesirable trait for some consumers [5, 15].

FIGURE 1Ripe fruits of (a) ‘Encore’ and (b) ‘Nadorcott’ mandarins on the tree.

**Figure:**
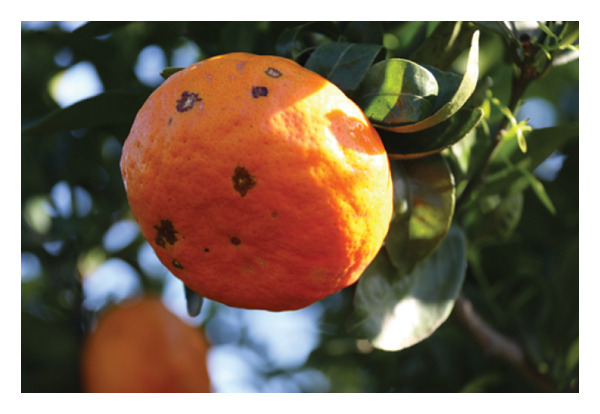
(a)

**Figure:**
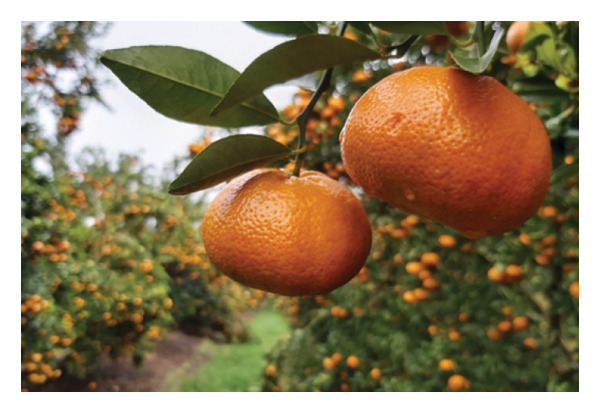
(b)

‘Nadorcott’, also known as ‘Afourer’ or ‘W. Murcott’, is a mandarin cultivar originating from a seedling of the ‘Murcott’ tangor, itself a hybrid between a true mandarin and a sweet orange (*C. reticulata* Blanco × *C. sinensis* Osbeck) [16]. ‘Nadorcott’ is also an intermediate fruit development cycle cultivar, directly rivalling ‘Encore’ in the fresh fruit market [17]. This cultivar is highly productive [16, 18, 19], although high yields can hinder tree development [20]. Unlike ‘Encore’, the fruits are not susceptible to preharvest rind‐stain but are more sensitive to long storage periods and conditions [7, 9, 21, 22]. Therefore, more care is required to maintain fruit quality [23] and avoid postharvest rind disorders, such as staining and pitting. Being self‐incompatible, it tends to produce very few or no seeds, when cultivated in isolation from other citrus cultivars. Its visual ‘perfection’ (Figure 1(b)) and seedlessness make it the preferred choice for major retailers and particularly appealing to consumers.

The external appearance of fruits and vegetables strongly influences consumer preferences [24–27]. Other attributes, such as texture, firmness [28–30] aroma and flavour, often shaped by previous experiences also affect the choice [28, 31–33]. Price and farming system likewise play important roles, with many consumers favouring organic options for health and environmental reasons [11, 34–36]. Familiarity with local brands or growers may also influence consumers’ preferences due to perceived quality or the desire to support the local economy [34, 35]. Additionally, convenience related to packaging and handling (e.g., precut or prepackaged fruits) can also influence preferences [37–40].

Major retailers often assume that consumers prioritize visual appearance, rejecting large quantities of fruit that do not meet strict aesthetic standards [31, 41, 42]. This reinforces and amplifies expectations about ‘ideal’ fruit appearance [31, 43] and leads to the systematic rejection of produce with minor defects regardless of internal quality [44, 45]. Such practices exacerbate food waste and increase the negative impact of farming on the economy and environmental sustainability [46–48] conflicting with global carbon‐neutrality goals [49]. In parallel, many new mandarin cultivars selected for productivity, appearance, transport resilience and shelf life are replacing older cultivars valued for superior flavour. In the Algarve, citrus producers report stringent retailer standards for peel defects that prevent marketing of otherwise edible fruit, causing large disposals and a decline in ‘Encore’ orchards; nonetheless, consumers familiar with ‘Encore’ continue to seek it. The practice of discarding products with imperfect appearance, like ‘Encore’, is unsustainable and should be avoided, truly understanding the preferences of the consumers, especially in the Algarve region and raising awareness among consumers and major retailers [31, 50, 51]. Evidence suggests that when consumers taste fruit with superior flavour, they may develop strong cultivar loyalty despite external imperfections. Accordingly, with his work, we aim to demonstrate that the internal quality of the fruit is not related to its external appearance, and that consumer choice based solely on appearance may lead to a low level of satisfaction, which may even result in reduced fruit consumption, with negative consequences for public health.

## 2. Materials and Methods

### 2.1. Fruit Sampling

The mandarins of the ‘Encore’ and ‘Nadorcott’ cultivars were harvested in the second week of March in southern Portugal and transported to the laboratory at the University of Algarve. For each cultivar, a sufficient quantity of fruit was selected to obtain two subsamples: one for organoleptic evaluation by a panel of tasters and the other for physicochemical analysis.

### 2.2. Organoleptic Evaluation

The organoleptic evaluation was conducted at the University of Algarve, in a dedicated room conforming to standard organoleptic testing conditions. Participants were seated individually in isolated booths, thereby avoiding possible perceptions of other tasters’ reactions. Water and sink were provided to rinse the mouth between tasting different samples.

#### 2.2.1. Characterization of the Participants on the Tasting Panel

**FIGURE 2:**
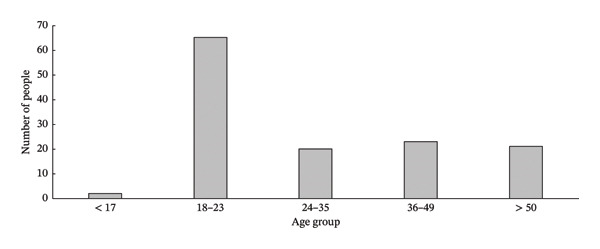
Age distribution of participants included in this study.

The tasting panel consisted of 131 participants from both genders, aged between 17 and over 50 (Figure 2). Participants were approached at random and invited to participate in the organoleptic tests. It was ensured that they were regular consumers of fruit, with no specific requirements in terms of social class or education. The inclusion of untrained tasters ensures a good representation of the consumer population, who typically do not have specialized sensory knowledge, thereby providing valuable information on how the average consumer perceives and experiences the fruit tested.

#### 2.2.2. Questionnaire

A digital platform (Google Forms) was used to conduct the tasting questionnaire, which was accessible to participants via personal devices such as smartphones. The questionnaire comprised four sections (Annex 1): (i) taster profile, (ii) fruits internal quality assessment, (iii) fruits external quality assessment and (iv) the final opinion.

The first section allowed participants to optionally indicate their name and select their age group from the following categories: < 17, 18–23, 24–35, 36–49 and > 50.

Participants received two samples, each one corresponding to a cultivar, without any prior knowledge of the distinctions between them. Each sample, ‘Encore’ (Figure 3(a)) and ‘Nadorcott’ (Figure 3(b)), comprised three fruit segments. In the second section, participants were queried about various aspects of internal quality, including internal appearance, texture, taste‐related factors (such as acidity and sweetness) and whether the seeds were perceived as bothersome. Additionally, their purchase intention was assessed based solely on internal quality.

FIGURE 3Fruit samples for internal quality, each with three fruit segments from three fruits: (a) ‘Encore’ and (b) ‘Nadorcott’.

**Figure:**
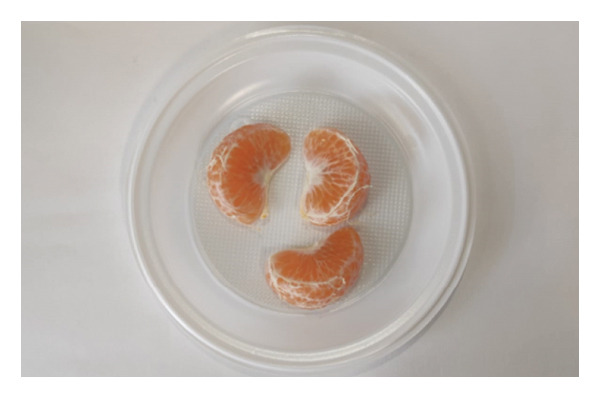
(a)

**Figure:**
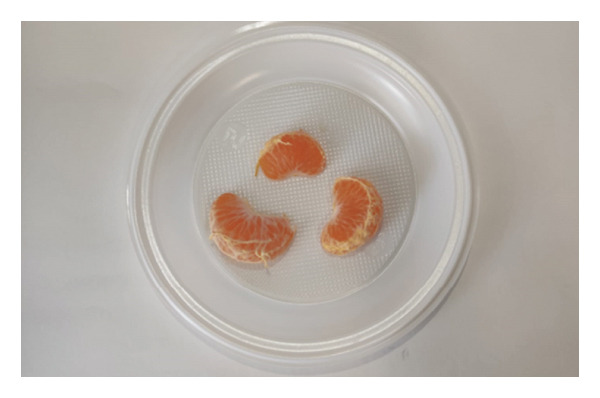
(b)

Following their responses to the second section, participants were then directed to evaluate the external appearance of the fruit in the third section. To this end, participants evaluated a representative sample of seven fruits from each cultivar: “Encore” (Figure 4(a)) and “Nadorcott” (Figure 4(b)). Regarding the “Encore” mandarin, fruits displaying the highest levels of epidermal damage were excluded, as these are typically rejected at the packing house and are therefore not available in the retail market. Different identifiers were used for each cultivar to prevent any influence from the previously assessed internal quality. Participants were asked about their intention to purchase based on the external quality alone.

FIGURE 4Fruit samples for external quality: (a) ‘Encore’ and (b) ‘Nadorcott’.

**Figure:**
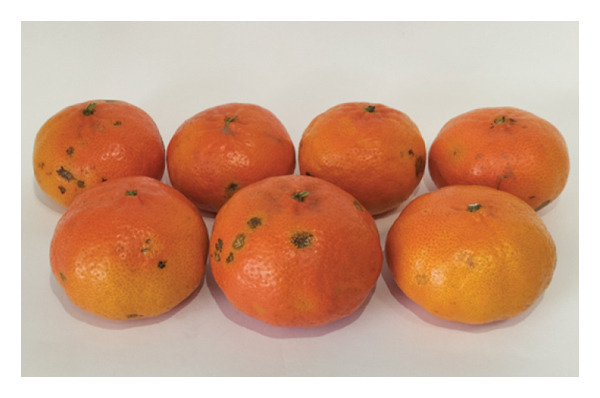
(a)

**Figure:**
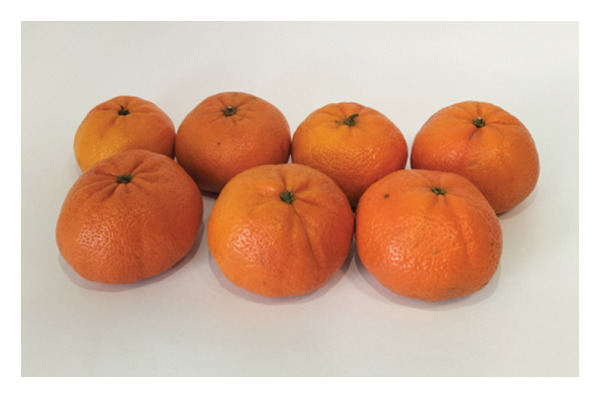
(b)

In the fourth section, the correspondence between the fruits assessed for internal quality in the second section and those assessed for external quality in the third section was revealed. Participants were then asked to answer the final question regarding their final purchase intention, considering both internal and external quality assessments. This approach was designed to provide a comprehensive understanding of participants’ preferences by integrating their evaluations of both the internal and external fruit attributes.

Responses in the second, third and fourth sections employed a five‐point hedonic scale ranging from 1 (Lowest) to 5 (Highest).

### 2.3. Physicochemical Analyses

In parallel with the tasting panel, the physical and chemical qualities of both cultivars were assessed. Initially, nondestructive analyses were performed, including weight, diameter and colour. The colour was measured with a colorimeter (Konica Minolta, Chroma Meter CR‐400), and the citrus colour index [CCI = (1000 × *a*)/(*L* × *b*)] was calculated, where *L*, *a* and *b* are the coordinates of the Hunter Lab colour space.

Subsequently, destructive analyses were performed. Peel thickness was determined by the average of two measurements taken at two points positioned at 90° between each other in the fruit’s equatorial zone. The TSS (°Brix) was measured with a refractometer (Atago, PAL‐BX|ACID1), at room temperature, while juice acidity was determined by titration with a 0.1 M sodium hydroxide (NaOH) solution. The maturation index (MI = soluble solids content/acidity) was then calculated.

### 2.4. Statistical Analysis and Writing

To analyse the questionnaire responses, concise descriptive statistics were used to summarise the results. To identify potential significant differences in responses between the ‘Encore’ and ‘Nadorcott’ cultivars, a nonparametric Kolmogorov–Smirnov test was applied, based on the comparison of median values. The choice of this test was driven by the nature of the variables, which adhered to an ordinal scale with only five levels. In addition, the variables did not meet the requirements of normality and symmetry requisite for employing Student’s t‐test for comparing means. To find out if there were any significant differences between the physicochemical fruit quality parameters, the parametric Student’s t‐test was used to compare the averages of two cultivars. All the tests were performed using the software IBM SPSS Statistics (Version 29.0).

A language‐based artificial intelligence model (ChatGPT) was used to support translations and grammatical revisions of parts of the text, with all authors providing subsequent scientific validation.

## 3. Results

### 3.1. External Quality

The results of the physicochemical fruit quality for both cultivars showed that the ‘Encore’ cultivar had larger fruits in terms of both weight (+81.3 g) and diameter (+13.3 mm) and also demonstrated a higher colour index (+5.5) (Table 1).

**TABLE 1:** Weight, diameter and citrus colour index (CCI) for ‘Encore’ and ‘Nadorcott’ mandarins, along with Student’s t‐test results and its significance (Sig.) values.

Cultivar	Weight (g)	Diameter (mm)	Citrus colour index (CCI)
‘Encore’	172.9 ± 2.3	76.8 ± 0.4	21.4 ± 0.3
‘Nadorcott’	91.6 ± 0.8	63.5 ± 0.2	20.2 ± 0.1
Student’s t‐test	−33.133	−30.837	−3.357
Sig.	< 0.001	< 0.001	< 0.001

*Note:* A significance value below 0.05 indicates statistical differences between the two cultivars.

Regarding the tasters’ responses to the organoleptic test for both external quality factors, appearance (Figure 5(a)) and purchase intention (Figure 5(b)), reveal a clear preference for ‘Nadorcott’, supported by clear statistical evidence. For external appearance, ‘Nadorcott’ had a median score of 5 (average of 4.46), while ‘Encore’ had a median score of 3 (average of 2.95). In terms of purchase intention based on external quality, ‘Encore’ showed a median score of 3 (average of 3.19), whereas ‘Nadorcott’ had a median of 4 (average of 4.25), statistically higher than that of ‘Encore’.

FIGURE 5Distribution of internal quality data for each factor, including its average (×), median (thicker lines), quartiles, interquartile range and outliers: (a) external appearance and (b) purchase intention (external). For factor (a): 1—I definitely don’t like it; 2—I don’t like it; 3—Neutral; 4—I like it; and 5—I definitely like it. For factor (b): 1—I certainly wouldn’t buy it; 2—I wouldn’t buy it; 3—Maybe I would buy it; 4—Would buy it; and 5—I would definitely buy it. Equal letters indicate the absence of statistical difference, according to the nonparametric Kolmogorov–Smirnov statistical test (*p* = 0.05).

**Figure:**
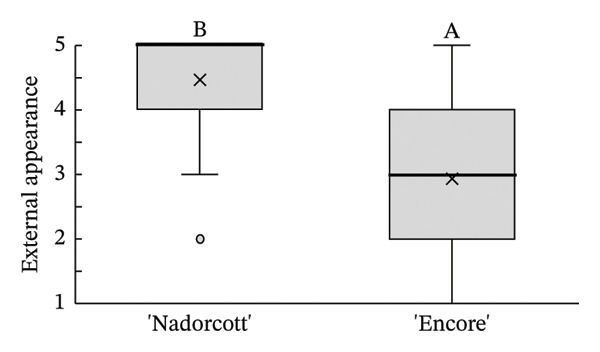
(a)

**Figure:**
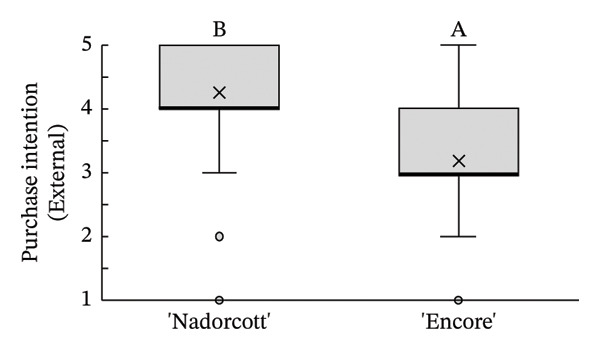
(b)

**FIGURE 6:**
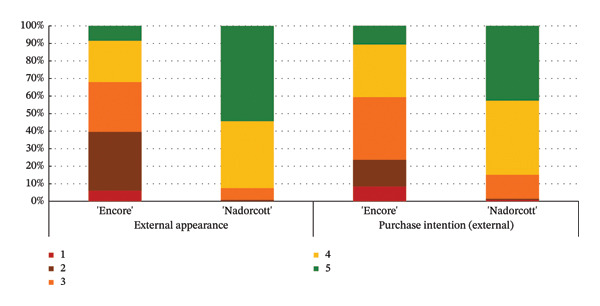
Percentage distribution of taster responses across external quality factors: external appearance and purchase intention (external). External appearance: 1—I definitely don’t like it; 2—I don’t like it; 3—Neutral; 4—I like it; and 5—I definitely like it. Purchase intention (external): 1—I certainly wouldn’t buy it; 2—I wouldn’t buy it; 3—Maybe I would buy it; 4—Would buy it; and 5—I would definitely buy it.

The distribution of taster responses across different levels for each external quality factor, external appearance and purchase intention (external), shows that 92% of the tasters responded that they ‘definitely liked’ or ‘liked’ the external appearance of the ‘Nadorcott’ mandarin, which is 60% higher than that for ‘Encore’ (Figure 6). Purchase intention (external) was based solely on the external appearance. 85% percent of the tasters indicated that they ‘would buy’ or ‘definitely buy’ ‘Nadorcott’, which is 44% more than for ‘Encore’. In contrast, 24% of respondents said they ‘would not buy’ or ‘certainly would not buy’ ‘Encore’, a percentage that is 22% higher than for ‘Nadorcott’.

### 3.2. Internal Quality

Concerning the physicochemical analyses of the fruit’s internal quality, the ‘Encore’ cultivar exhibited a lower rind thickness, a 17% higher TSS content than that of the ‘Nadorcott’, combined with a 39% lower acidity, resulting in a 51% higher MI (Table 2).

**TABLE 2:** Rind thickness (RT), total soluble solids (TSS), acidity and maturation index (MI) for each cultivar, along with Student’s t‐test results and its significance (Sig.) values.

Cultivar	RT (mm)	TSS (°Brix)	Acidity (g.100 mL^−1^)	MI
‘Encore’	2.21 ± 0.02	13.8 ± 0.1	0.88 ± 0.01	16.0 ± 0.2
‘Nadorcott’	2.69 ± 0.03	11.5 ± 0.1	1.44 ± 0.01	8.06 ± 0.04
Student’s t‐test	13.698	−25.726	25.434	−38.313
Sig.	< 0.001	< 0.001	< 0.001	< 0.001

*Note:* A significance value below 0.05 indicates statistical differences between the two cultivars.

The distribution of taster’s responses across internal quality factors, including internal appearance (Figure 7(a)), aroma (Figure 7(b)), texture (Figure 7(c)), sweetness (Figure 7(d)), acidity (Figure 7(e)), off‐flavour presence (Figure 7(f)), general flavour (Figure 7(g)), the nuisance level of the seeds (Figure 7(h)) and purchase intention regarding internal appearance (Figure 7(i)), indicates a preference for ‘Encore’ among tasters, with higher ratings for internal appearance, aroma, sweetness, acidity, general taste and purchase intention (internal). Responses show a level of indifference towards texture and the presence of off‐flavours. The only discernible preference for ‘Nadorcott’ emerges in relation to the presence of seeds, suggesting discomfort among tasters with the seed content in ‘Encore’. This trend was statistically validated by the Kolmogorov–Smirnov test, confirming that tasters consistently preferred ‘Encore’ in terms of its internal quality.

FIGURE 7Distribution of internal quality data for each factor, including its average (×), median (thicker lines), quartiles, interquartile range and outliers: (a) Internal appearance, (b) aroma, (c) texture, (d) sweetness, (e) acidity, (f) off‐flavour, (g) general flavour, (h) do seeds bother? and (i) purchase intention (internal). For factors (a), (b), (c), (d), (e) and (g): 1—I definitely don’t like it; 2—I don’t like it; 3—Neutral; 4—I like it; and 5—I definitely like it. For factor (f): 1—Extremely unpleasant; 2—Unpleasant; 3—Slightly unpleasant; 4—Present but doesn’t bother; and 5—Present but really don’t bother. For factor (h): 1—They are very bothersome; 2—They bother; 3—Neutral; 4—They don’t bother; and 5—They really don’t bother. For factor (i): 1—I certainly wouldn’t buy it; 2—I wouldn’t buy it; 3—Maybe I would buy it; 4—Would buy it; and 5—I would definitely buy it. Equal letters indicate the absence of statistical difference, according to the nonparametric Kolmogorov–Smirnov statistical test (*p* = 0.05).

**Figure:**
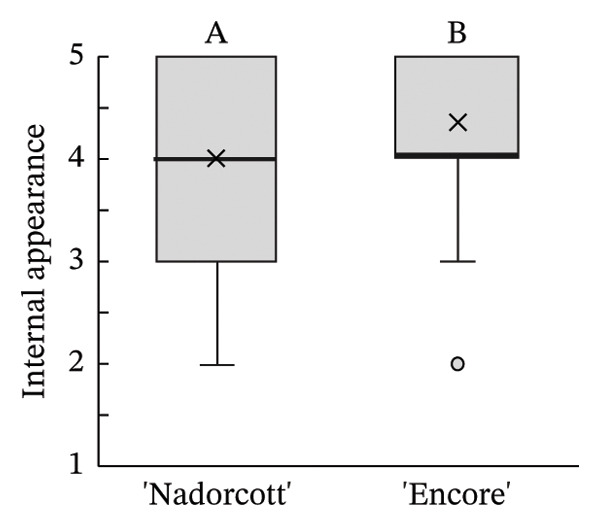
(a)

**Figure:**
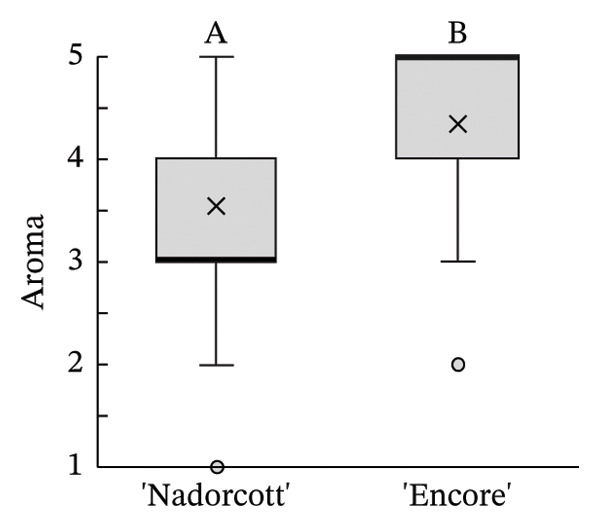
(b)

**Figure:**
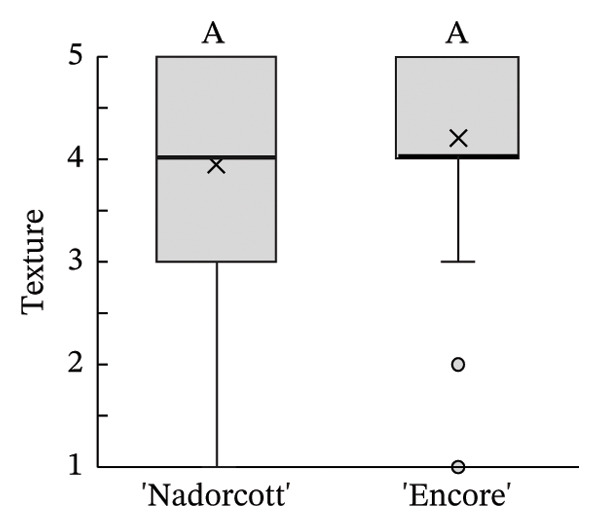
(c)

**Figure:**
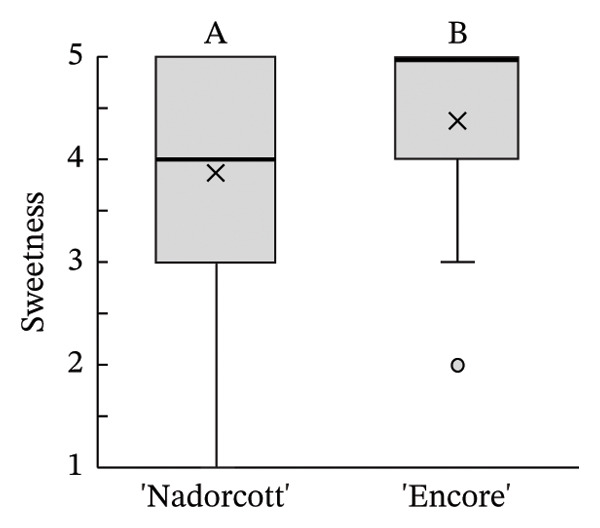
(d)

**Figure:**
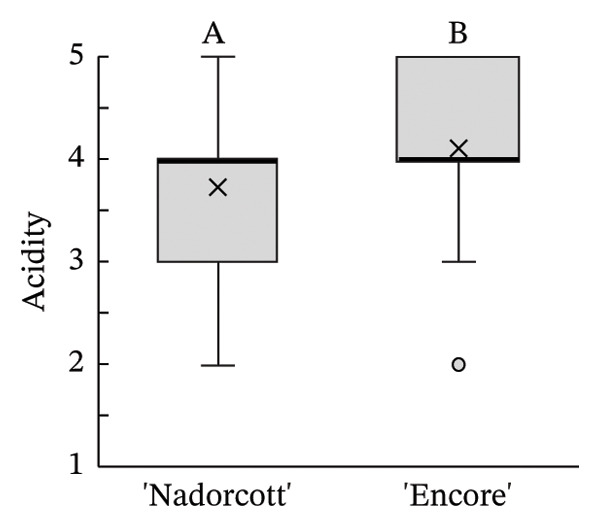
(e)

**Figure:**
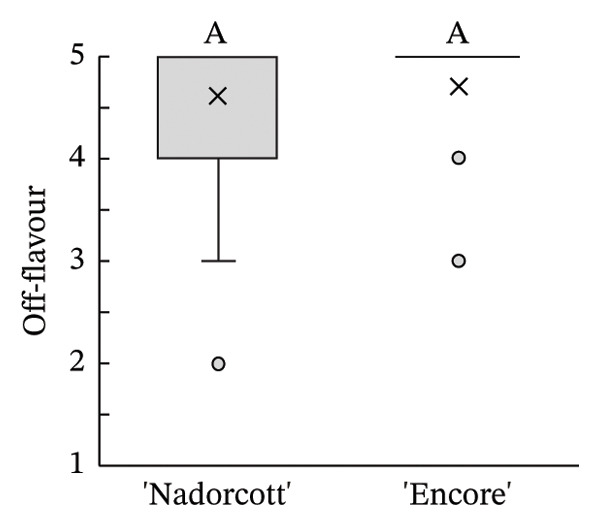
(f)

**Figure:**
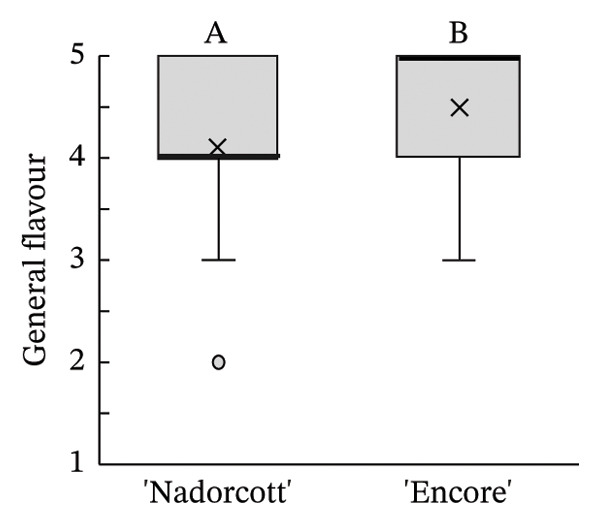
(g)

**Figure:**
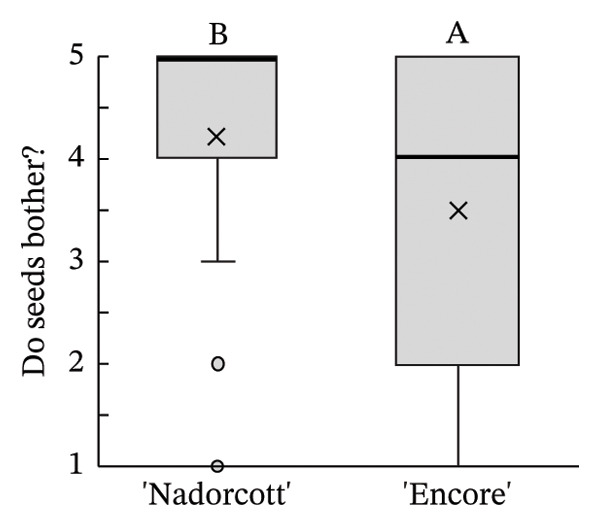
(h)

**Figure:**
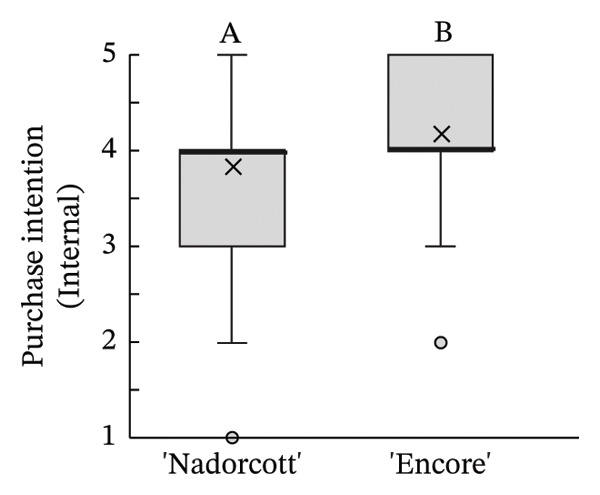
(i)

**FIGURE 8:**
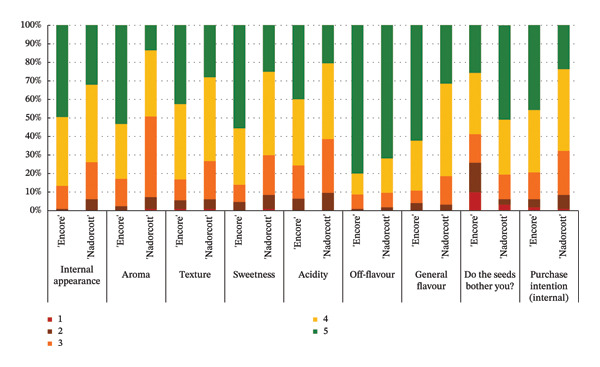
Percentage distribution of taster responses across internal quality factors: internal appearance, texture, sweetness, acidity, off‐flavour, general flavour, do seeds bother? and purchase intention (internal). Internal appearance, aroma, texture, sweetness, acidity and general flavour: 1—I definitely don’t like it; 2—I don’t like it; 3—Neutral; 4—I like it; and 5—I definitely like it. Off‐flavour: 1—Extremely unpleasant; 2—Unpleasant; 3—Slightly unpleasant; 4—Present but doesn’t bother; and 5—Present but really don’t bother. Do seeds bother?: 1—They are very bothersome; 2—They bother; 3—Neutral; 4—They don’t bother; and 5—They really don’t bother. Purchase intention (internal): 1—I certainly wouldn’t buy it; 2—I wouldn’t buy it; 3—Maybe I would buy it; 4—Would buy it; and 5—I would definitely buy it.

The taster’s response percentages for each of the internal quality factors seem to have consistently favoured the ‘Encore’ over ‘Nadorcott’ (Figure 8). One of the factors that stands out is aroma, with 83% of tasters expressing they ‘liked’ or they ‘definitely liked’ ‘Encore’, a 34% increase compared to ‘Nadorcott’. Following the aroma, sweetness also stood out, with 86% of tasters expressing a liking for ‘Encore’, which is 16% higher than for ‘Nadorcott’. In the overall taste, ‘Encore’ received 8% more ‘like’ or ‘definitely like’ responses compared to ‘Nadorcott’. Regarding the intention to purchase based on internal quality alone, tasters showed a preference for ‘Encore’, with nearly 80% expressing a willingness to buy, a 12% percentage higher than that observed for ‘Nadorcott’. Nevertheless, the prevalence of seeds in ‘Encore’, consistently abundant, appeared to be more of an issue for tasters compared to ‘Nadorcott’, where seeds were almost ever absent or minimal. Over 25% of tasters expressed dissatisfaction with the seeds in ‘Encore’, describing them as ‘they are very bothersome’ or stating that ‘they bother’, a percentage 20% higher than in ‘Nadorcott’. Conversely, nearly 60% of tasters in ‘Encore’ did not find the seeds too bothersome, responding with ‘they don’t bother’ or ‘they really don’t bother’, but still representing a 22% lower percentage compared to ‘Nadorcott’.

### 3.3. Ultimate Purchase Intention

**FIGURE 9:**
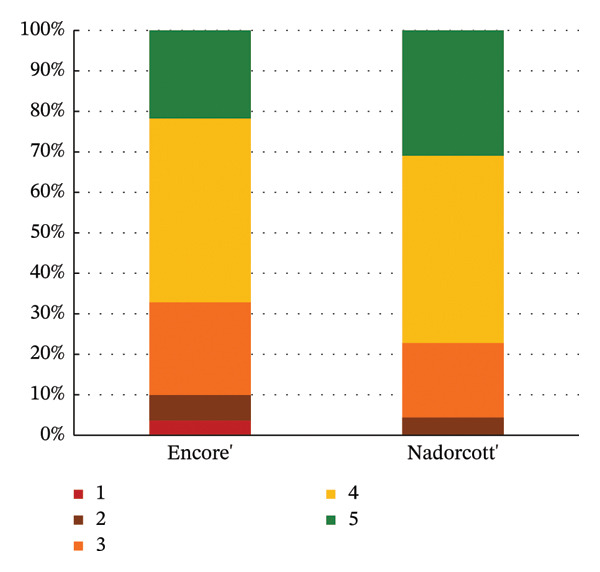
Percentage distribution of taster responses across the final purchase intention. Final purchase intention: 1—I certainly wouldn’t buy it; 2—I wouldn’t buy it; 3—Maybe I would buy it; 4—Would buy it; and 5—I would definitely buy it.

**FIGURE 10:**
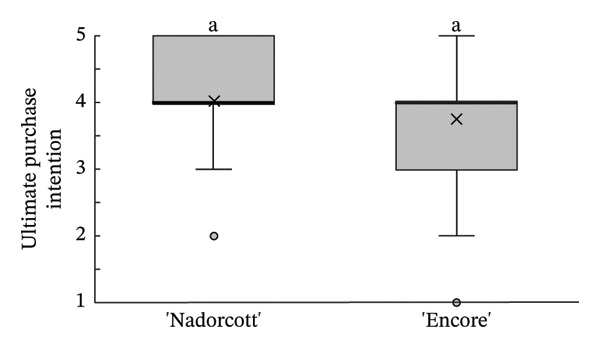
Distribution of final purchase intention responses, including its median (thicker lines), quartiles, interquartile range and outliers: 1—I certainly wouldn’t buy it; 2—I wouldn’t buy it; 3—Maybe I would buy it; 4—Would buy it; and 5—I would definitely buy it. Equal letters indicate the absence of statistical difference, according to the nonparametric Kolmogorov–Smirnov statistical test (*p* = 0.05).

In the final purchase intention question, approximately 77% of tasters indicated their intent to ‘buy’ or ‘definitely buy’ ‘Nadorcott’, which is 10% higher than for ‘Encore’ (Figure 9). The distribution of response values (Figure 10) implies a subtle inclination towards ‘Nadorcott’, but it is not statistically significant.

## 4. Discussion

In the external physicochemical analyses of this study (Table 1), ‘Encore’ had higher fruit weight (+47%), greater diameter (+18%) and higher colour index (+6%) when compared to ‘Nadorcott’. Still, it seems this was not enough to counterbalance the bad visual impact caused by the rind‐stain disorder in ‘Encore’, and the tasters preferred the external appearance of the ‘Nadorcott’ mandarin (Figure 5), with 92% of them indicating that they ‘definitely liked’ or ‘liked’ its appearance, 60% more than those who responded the same about ‘Encore’ (Figure 6). The dislike of the external appearance of the ‘Encore’ mandarin negatively impacted the purchase intention in the ‘blind’ evaluation of the external appearance. A notable 24% of the tasters responded negatively (‘would definitely not buy’ or ‘would not buy’) to the possibility of purchasing ‘Encore’ mandarins based on its visual appearance, a 22% percentage higher than for ‘Nadorcott’ (Figure 6). This confirms the critical importance of external appearance in consumer decision‐making, reaffirming that the presence of defects in fruit peel has a negative impact on consumer preference, as is widely recognized [25, 52, 53].

On the other hand, the superior internal physicochemical characteristics of ‘Encore’ seemed to be aligned with the tasters’ preferences in the ‘blind’ evaluation of internal quality. The internal physicochemical analyses suggest that ‘Encore’ has a higher MI, which results from a better balance between sweetness (TSS) and acidity (Table 2). In ‘Encore’, the TSS was nearly 14°Brix, a value that is even higher than that reported by other authors [21]. For ‘Nadorcott’, the TSS was of approximately 11.5°Brix, similar to what has been observed by other authors on day 0 of storage [22] and lower from what can be expected from late orange cultivars [54]. Other studies have characterized ‘Encore’ as one of the citrus cultivars with higher TSS content [10]. ‘Encore’ also had lower acidity, and, combined with its higher TSS, this led to a 51% higher MI than for ‘Nadorcott’. These results are consistent with other studies that agree that sweetness and acidity are crucial parameters in the overall perception of flavour, especially in citrus fruits [4, 55], and that the consumers tend to prefer less acidic fruits [56].

In addition, consumers clearly preferred the aroma of ‘Encore’. It is possible that the specific combination of organic compounds that form ‘Encore’s’ aroma is more appealing than that of ‘Nadorcott’. Other studies have found that, when compared to other mandarins, ‘Nadorcott’ did not stand out in terms of flavour or aroma intensity, being only moderately appreciated [5]. Citrus aroma is mostly determined by volatile compounds such as esters, aldehydes, alcohols, ketones and hydrocarbons [57]. However, other factors such as phenols, sugars, acids and postharvest conditions can also influence the fruit’s aroma. Mandarins are known to contain high levels of various volatile compounds, including limonene, linalool, γ‐terpinene, β‐myrcene, α‐pinene and octanal, in both fruits [58] and flowers [59]. The volatile content can be influenced by factors such as rootstock [60], diseases [61] and others. Volatile compounds and phenols can impact the commercial value, especially if related to beneficial effects on human health [62–66].

The internal appearance of the fruit, based on the preferred look of the fruit segments, was also favoured in ‘Encore’ (Figure 7). The visual appearance of mandarin segments also plays a role in consumer preference. Other studies have shown that segment appearance can vary significantly, directly impacting the perceived quality and freshness of the fruit. Segment colour is often the first attribute noticed, with vibrant, deep orange segments being more attractive and associated with sweetness and ideal ripeness [67, 68]. The presence, adherence and colour of the albedo can significantly influence the appearance of mandarin segments. A thick, strongly adhered or discoloured albedo, such as yellowish or greenish, can negatively affect the visual appeal, making the segments seem less fresh or appetizing [68, 69]. Segment size also influences the perception, as larger, more uniform segments are seen as juicier and more satisfying. In this case, ‘Encore’ segments were larger, which is due to a higher fruit size (Table 1). Still, other authors have observed that some consumers prefer smaller tangerine segments [70]. Additionally, well‐shaped segments with smooth contours and symmetry enhance visual appeal, making them more desirable for consumers who value aesthetics and ease of consumption [71]. The texture of the segments in the mouth is a critical quality attribute in consumer preference [72, 73], with consumers preferring ‘juicy’ and ‘not fibrous’ mandarins [74]. In our case, texture was equally appreciated in both cultivars, with a median rating of 4 (‘I like it’). Other authors found that ‘Nadorcott’ had a notably softer texture, whereas ‘Ortanique’ tangor [(*C*. × *reticulata*) × (*C*.  × *sinensis*)] was associated with a tougher texture that made chewing and swallowing more difficult, characteristics that consumers view as undesirable [75]. The only internal quality factor where tasters clearly preferred ‘Nadorcott’ over ‘Encore’ was the presence of seeds. This was expected and in agreement with other studies that found that consumers prefer fruit without seeds, making it a key factor in their consumption choices [76]. In addition, many consumers seek products that offer fresh‐like quality while being convenient to consume, driving the development of new technologies to create ready‐to‐eat mandarins [77] and other citrus fruit segments [78], associated with seedless products. However, it is interesting to notice that in the present study, despite the elevated seed presence in ‘Encore’, the superior ratings for aroma, sweetness and acidity led to a preference to buy it when evaluating the internal quality.

Previous studies already found out that consumers did not strongly prefer ‘Nadorcott’, and it was generally outperformed by other mandarin cultivars [5]. However, the results of this study highlight the significant impact that internal quality have on consumer preferences. From the ‘blind’ external appearance evaluation, the spots on the peel of ‘Encore’ led tasters to prefer to buy ‘Nadorcott’, with 44% more tasters indicating they would ‘buy’ or ‘definitely buy’ it. However, this purchase intent completely shifted when the selection criterion was the internal appearance. ‘Encore’ saw a 39% increase in ‘buy’ or ‘definitely buy’ responses compared to its external appearance, while ‘Nadorcott’ experienced a 17% drop. This shift in the purchase intent, in the last section of the questionnaire, resulted in the final overall intention being statistically similar for both cultivars.

The initial preference for ‘Nadorcott’ driven by its superior external appearance confirms the importance of first impressions. Consumers more and more often rely on visual cues as a quick assessment of quality, especially in a competitive market [79, 80] where the standards have been biased by this feedback loop that shapes and amplifies consumer expectations of frit visual appearance [31, 43]. However, the subsequent shift in the favour of ‘Encore’ when internal qualities were considered reveals that deeper attributes, such as flavour and texture, can override initial judgements [81]. This suggests that while an attractive exterior may draw consumers in, the intrinsic qualities of a product ultimately carry significant weight in determining long‐term purchasing decisions. Therefore, for producers and retailers, it is essential to balance both external appeal and internal quality to ensure sustained consumer interest and loyalty. In addition, implementing consumer information strategies could support the transition to a more sustainable future, particularly regarding food waste reduction and production practices.

This work helps to explain why some citrus cultivars with poor external appearance remain in the market, particularly in local markets where consumers familiar with these cultivars predominate.

## 5. Conclusion

Defects in the skin of ‘Encore’, a result of a physiological issue inherent to the cultivar, led tasters to favour ‘Nadorcott’ based on its external appearance. However, once the tasters tasted the fruits, their preference shifted significantly towards ‘Encore’. The aroma, sweetness, acidity and overall flavour of ‘Encore’ was notably more appreciated. Only when seeds were present did tasters prefer ‘Nadorcott’, which has few or no seeds compared to ‘Encore’. Both the ‘Encore’ and ‘Nadorcott’ mandarins were positively evaluated by tasters, which reflects the good acceptance of both cultivars by the market.

Implementing policies that improve consumer knowledge of available products could promote more sustainable consumption and increase satisfaction. Providing simple label information on fruit sweetness and acidity would enable consumers to select fruit based on flavour, without requiring prior knowledge of cultivars or harvest seasons.

## Funding

This work is funded by the National Funds through FCT–Foundation for Science and Technology under Project UID/05183/2025.

## Conflicts of Interest

The authors declare no conflicts of interest.

## Data Availability

The data that support the findings of this study are available from the corresponding author upon reasonable request.
